# Which is the optimal immunotherapy for advanced squamous non-small-cell lung cancer in combination with chemotherapy: anti-PD-1 or anti-PD-L1?

**DOI:** 10.1186/s40425-018-0427-6

**Published:** 2018-12-03

**Authors:** Yaxiong Zhang, Huaqiang Zhou, Li Zhang

**Affiliations:** 1 Department of Medical Oncology, Sun Yat-sen University Cancer Center, 651 Dongfeng Road East, Guangzhou, Guangdong China; 2 State Key Laboratory of Oncology in South China, 651 Dongfeng Road East, Guangzhou, Guangdong China; 3 Collaborative Innovation Center for Cancer Medicine, 651 Dongfeng Road East, Guangzhou, Guangdong China

**Keywords:** NSCLC, Squamous, Anti-PD-1, Anti-PD-L1, Pembrolizumab, Atezolizumab

## Abstract

Recent randomized phase III trials (KEYNOTE-407 and IMpower131) reported that adding anti-programmed death (ligand) 1 (anti-PD-(L)1) antibodies in combination with taxane-platinum improve the therapeutic efficacy for advanced squamous non-small-cell lung cancer (NSCLC). However, there is no head-to-head comparison of pembrolizumab (anti-PD-1) plus chemotherapy vs. atezolizumab (anti-PD-L1) plus chemotherapy. Therefore, we performed an indirect comparison to explore the optimal choice of anti-PD-(L)1 treatment for advanced squamous NSCLC in combination with chemotherapy. The clinical outcomes were overall survival (OS), progression-free survival (PFS), objective response rate (ORR) and adverse event (AE). For overall patients, pembrolizumab had significantly superior OS (hazard ratio (HR) with 95% confidence interval, 0.67, 0.47–0.94; *P* = 0.02) and numerically better PFS (HR, 0.79, 0.60–1.04; *P* = 0.10) than atezolizumab, while they had similar ORR, all cause AE and grade 3–5 AE. For PD-L1 high patients, pembrolizumab and atezolizumab showed similar OS and PFS. However, for PD-L1 low/negative patients, pembrolizumab had superior OS (HR, 0.43, 0.24–0.76; *P* <  0.01/ HR, 0.74, 0.40–1.38; *P* = 0.35) and better PFS (HR, 0.80, 0.51–1.26; *P* = 0.33/ HR, 0.46, 0.28–0.75; *P* <0.01) than atezolizumab. Our analysis raises the hypothesis that anti-PD-1 antibody therapy in combination with chemotherapy may have superior efficacy compared to anti-PD-L1 antibody combination for patients with PD-L1 low/negative advanced squamous NSCLC.

## Background

Adenocarcinoma and squamous carcinoma are two most common histological subtype of advanced non-small-cell lung cancer (NSCLC). Patients with lung adenocarcinoma whose tumor harbor specific gene mutations, such as epidermal growth factor receptor (EGFR) mutation or anaplastic lymphoma kinase (ALK) fusion, derive significant benefit from targeted agents, tyrosine kinase inhibitors (TKIs), and have better prognosis [1]. However, this advancement has not been achieved in squamous NSCLC given the lack of efficacy and there are currently no approved targeted agents for squamous NSCLC [1]. The standard treatment for advanced squamous NSCLC includes platinum-based doublet chemotherapy, such as taxane-platinum combination, which has poor efficacy [2]. Therefore, we still need to explore a better therapeutic regimen for advanced squamous NSCLC. Recently, a randomized phase III trial (KEYNOTE-407) reported that adding pembrolizumab, an anti-programmed death 1 (anti-PD-1) antibody, in combination with carboplatin plus paclitaxel/nab-paclitaxel decreases the mortality risk for advanced squamous NSCLC [3]. Meanwhile, another randomized phase III study (IMpower131) showed that combined carboplatin plus nab-paclitaxel with atezolizumab, an anti-programmed death ligand 1 (anti-PD-L1) antibody, also improved the therapeutic efficacy for those patients [4]. However, there is no head-to-head comparison of pembrolizumab plus chemotherapy vs. atezolizumab plus chemotherapy. Therefore, we performed an indirect comparison of KEYNOTE-407 and IMpower131 to explore the optimal choice of anti-PD-(L)1 treatment for advanced squamous NSCLC in combination with chemotherapy.

## Methods

The clinical outcomes for our study were overall survival (OS), progression-free survival (PFS), objective response rate (ORR) and adverse event (AE). Data of OS and PFS were extracted as hazard ratio (HR) and its 95% confidence interval (CI), while data of ORR and AE were extracted as risk ratio (RR) and its 95% CI. All of above data were derived from KEYNOTE-407 and IMpower131. HR and RR represented pembrolizumab vs. atezolizumab. Based on the assumption that there is no significant treatment efficacy of carboplatin plus paclitaxel in comparison to carboplatin plus nab-paclitaxel for advanced squamous NSCLC, [5] we calculated the adjusted indirect comparison using the following formulas as previously described [6]. The log HR of the adjusted indirect comparison for arm A (pembrolizumab plus chemotherapy) vs. arm B (atezolizumab plus chemotherapy) was linked by arm C (chemotherapy), which was estimated by *log HR*_*AB*_ =  *log HR*_*AC*_ −  *log HR*_*BC*_, and its standard error (SE) for the log HR was $SE\;\left(\mathit{\log}\;{HR}_{AB}\right)=\sqrt{\  SE\;{\left(\mathit{\log}\;{HR}_{AC}\right)}^2+ SE\;{\left(\mathit{\log}\;{HR}_{BC}\right)}^2}$. RR was evaluated similarly as above formulas. HR <1 or RR> 1 standed for pembrolizumab had longer PFS/OS or better ORR/less AE than atezolizumab incombination with chemotherapy. A statistical test with *P*-value≤0.05 was considered as significant.

## Results

**Fig. 1:**
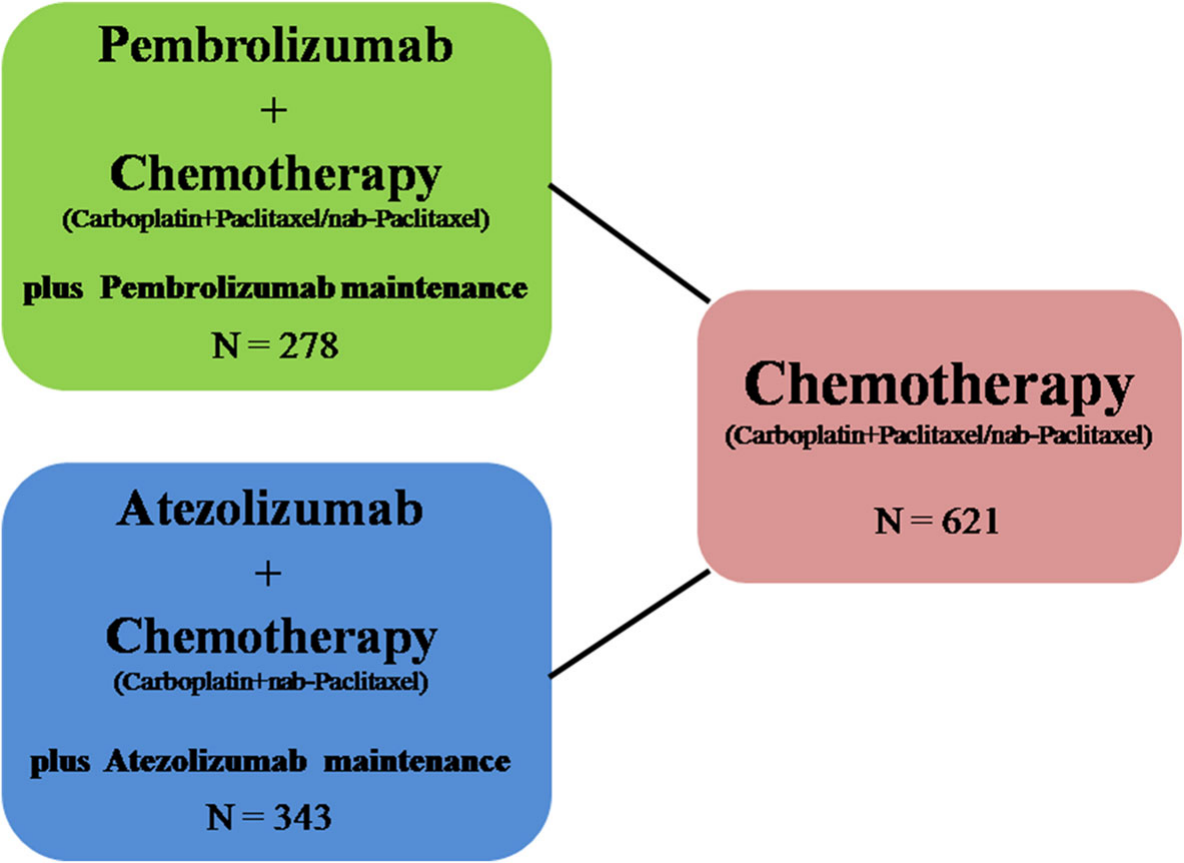
Diagram of the indirect comparison between pembrolizumab plus chemotherapy vs. atezolizumab plus chemotherapy for advanced squamous non-small-cell lung cancer. Solid lines between treatment regimens represented the existence of direct comparisons. *N* = enrolled patient number

Table 1 summarized study design, baseline characteristics and available endpoints of the trials in detail. We compared therapeutic efficacy and AE between pembrolizumab (*N* = 278) and atezolizumab (*N* = 343) in combination with chemotherapy as the first-line treatment of advanced squamous NSCLC (Fig. 1). The HR, RR and CI in the result part were calculated from our analysis and not from the above trials. For overall patients, pembrolizumab had significantly superior OS (HR, 0.67; 95% CI, 0.47–0.94; *P* = 0.02) and numerically better PFS (HR, 0.79; 95% CI, 0.60–1.04; *P* = 0.10) than atezolizumab, while they had similar ORR, all cause AE and grade 3–5 AE (Table 2). For PD-L1 high patients, pembrolizumab and atezolizumab showed similar OS and PFS, while pembrolizumab had significantly superior OS (HR, 0.43; 95% CI, 0.24–0.76; *P* <0.01) and numerically better PFS (HR, 0.80; 95% CI, 0.51–1.26; *P* = 0.33) than atezolizumab for PD-L1 low patients. Furthermore, pembrolizumab showed significantly longer PFS (HR, 0.46; 95% CI, 0.28–0.75; *P* <0.01) compared with atezolizumab for PD-L1 negative patients (Table 2).

**Table 1:** Baseline characteristics and available endpoints about the trials of KEYNOTE-407 and IMpower 131

Items	KEYNOTE-407		IMpower 131	
Baseline Characteristics	Pembro + CP/CnP (*N* = 278)	Placebo + CP/CnP (*N* = 281)	Atezo + CnP (*N* = 343)	CnP (*N* = 340)
Age, median (range), years	65.0 (29–87)	65.0 (36–88)	65 (23–83)	65 (38–86)
Sex, male, n (%)	220 (79.1)	235 (83.6)	279 (81)	278 (82)
Race, Asian, n (%)	54 (19.4)	52 (18.5)	41 (12)	37 (11)
ECOG PS, 0, n (%)	73 (26.3)	90 (30.0)	115 (34)	110 (32)
Former/current smoker, n (%)	256 (92.1)	262 (93.2)	311 (91)	216 (93)
PD-L1 expression^a^, n (%)				
High	73 (26.3)	73 (26.0)	53 (15)	48 (14)
Low	103 (37.1)	104 (37.0)	129 (38)	121 (36)
Negative	95 (34.2)	99 (35.2)	160 (47)	171 (50)
Endpoints				
Median follow-up (months)	7.8		17.1	
OS (months), HR (95%CI, *P*)	15.9 vs. 11.3; 0.64 (0.49–0.85, *P* = 0.0008)		14.0 vs. 13.9 0.96 (0.78–1.18, *P* = 0.6931)	
PFS (months), HR (95%CI, *P*)	6.4 vs. 4.8 0.56 (0.45–0.70, *P* <0.0001)		6.3 vs. 5.6 0.71 (0.60–0.85, *P* = 0.0001)	
ORR (%)	59.4 vs. 38.0		49.3 vs. 41.2	
All cause AEs (%)	98.2 vs. 97.9		99.4 vs. 97.0	
Grade 3–5 AEs (%)	69.8 vs. 68.2		82.5 vs. 70.1	

*Pembro* Pembrolizumab, *CP* carboplatin and paclitaxel, *CnP* carboplatin and nab-paclitaxel, *Atezo* Atezolizumab, *OS* overall survival, *PFS* Progression-free survival, *ORR* objective response rate, *AEs* Adverse Events, *HR* hazard ratio, *CI* confidence interval

^a^PD-L1 expression evaluation, KEYNOTE-407: The PD-L1 expression was assessed using the PD-L1 IHC 22C3 pharmDx assay, then determined by the Tumor Proportion Score (TPS) and classified into TPS < 1%, TPS 1 to 49% and TPS ≥50%. IMpower131: PD-L1 expression was evaluated using the VENTANA SP142 IHC assay. TC3 or IC3 (high) = TC ≥ 50% or IC ≥ 10% PD-L1+; TC1/2 or IC1/2 (low) = TC ≥ 1% and < 50% or IC ≥ 1% and < 10% PD-L1+; TC0 and IC0 (negative) = TC and IC < 1% PD-L1+. IC, tumor-infiltrating immune cell; TC, tumor cell

**Table 2:** Indirect comparison of pembrolizumab plus chemotherapy vs. atezolizumab plus chemotherapy for advanced squamous non-small-cell lung cancer

Item	Statistical analysis			
	HR / RR^a^ (95%CI)			*P*-value
Overall				
OS	**0.67**	0.47	0.94	**0.02**
PFS	0.79	0.60	1.04	0.10
Overall				
ORR	1.26	0.93	1.72	0.14
All cause AE	0.98	0.95	1.01	0.25
Grade 3–5 AE	0.87	0.76	1.01	0.06
PD-L1 High^b^				
OS	1.09	0.52	2.28	0.82
PFS	1.55	0.84	2.84	0.16
PD-L1 Low^c^				
OS	**0.43**	0.24	0.76	**< 0.01**
PFS	0.80	0.51	1.26	0.33
PD-L1 Negative^d^				
OS	0.74	0.40	1.38	0.35
PFS	**0.46**	0.28	0.75	**< 0.01**

Abbreviations: *CI* confidence interval, *HR* Hazard ratio, *RR* Risk ratio, *OS* overall survival, *PFS* progression-free survival, *ORR* objective response rate, *AE* adverse event, *PD-L1* programmed death ligand 1. A statistical test with *P*-value ≤ 0.05 was considered as significant

In IMpower131, PD-L1 expression was scored by immunohistochemistry (SP142 assay) in tumor cells (as percentage of PD-L1-expressing tumor cells ≥50%, TC3; ≥5% and < 50%, TC2; ≥1% and < 5%, TC1 and < 1%, TC0) and tumor-infi ltrating immune cells (as percentage of tumor area:≥10%, IC3; ≥5% and < 10%, IC2; ≥1% and < 5%, IC1; and < 1%, IC0). In KEYNOTE-407, PD-L1 expression was scored by immunohistochemistry (22C3 assay) in tumor cells (as percentage of PD-L1-expressing tumor cells TPS ≥50%, ≥1% and <50%, and < 1%)

^a^HR is used for OS and PFS evaluation, RR is used for ORR and AE evaluation

^b^PD-L1 High is defined as TC3 or IC3 in IMpower131, TPS ≥50% in KEYNOTE-407

^c^PD-L1 Low is defined as TC1/2 or IC1/2 in IMpower131, TPS ≥1% and < 50% in KEYNOTE-407

^d^PD-L1 Negative is defined as TC0 and IC0 in IMpower131, TPS < 1% in KEYNOTE-407

## Discussion

According to this indirect comparison, we found pembrolizumab plus chemotherapy seemed to be superior in terms of OS and PFS compared to atezolizumab plus chemotherapy, most notable in PD-L1 low/negative subgroup of patients. Not surprisingly, both of pembrolizumab and atezolizumab showed similar efficacy in PD-L1 high patients. Theoretically, PD-1 antibody can bind to PD-1 protein on T cells, so it will block the binding of PD-1 to PD-L1 and PD-L2 at the same time, while PD-L1 antibody can only interact with PD-L1, so it will only block the binding of PD-1 to PD-L1. Therefore, T cells might still be inhibited by the interaction between PD-1 and PD-L2 using anti-PD-L1 treatment [7]. For PD-L1 high patients, Anti-PD-L1 and Anti-PD-1 treatment might be effective similarly, because PD-L1 expression might be dominant for those patients. However, for PD-L1 low/negative patients, the expression spectrum of immunological molecule might be complicated, such as PD-L2 expression enhancement. As a result, Anti-PD-L1 treatment might not be enough compared with Anti-PD-1 treatment for PD-L1 low/negative patients.

The major limitation of this study was the limited follow-up time for KEYNOTE-407 and IMpower131, so that we used relative variables (HR and RR) instead of absolute value (median survival time) for analyses to lower the bias. Besides, the proportion of PD-L1 high patients was slightly higher in KEYNOTE-407, while the proportion of PD-L1 negative patients was slightly higher in IMpower131, both in experimental group and control group. It might cause imbalance of the patient population which affected the comparability of this indirect comparison. Moreover, PD-L1 expression was scored by SP142 assay in IMpower131, while it was scored by 22C3 assay in KEYNOTE-407, thus might have influence on PD-L1 level evaluation. Recent studies demonstrated the percentage of PD-L1-stained tumor cells was highly comparable among 22C3, 28–8 and SP263 PD-L1 assays, while SP142 assay exhibited fewer stained tumor cells, [8, 9] which was in accordance with the proportion of PD-L1 level population in KEYNOTE-407 (higher PD-L1 high patients) and IMpower131 (higher PD-L1 negative patients). To some extent, it proved that the overall patient population between KEYNOTE-407 and IMpower131 was comparable. But it should still be cautious to interpret the subgroup analysis stratified by PD-L1 level. After all, our study was an indirect comparison analysis, which might compromise the evidence level.

## Conclusions

These limitations aside, our study firstly compared pembrolizumab plus chemotherapy and atezolizumab plus chemotherapy for advanced squamous NSCLC and found the former seemed to be superior in terms of OS and PFS than the latter, especially in PD-L1 low/negative patients. Our analysis provides a hint that anti-PD-1 antibody might have superior efficacy compared to anti-PD-L1 antibody in combination with chemotherapy for patients with PD-L1 low/negative advanced squamous NSCLC. Additional studies are warranted to confirm this.
